# Lignin polymerization: towards high-performance materials

**DOI:** 10.1039/d4cs01044b

**Published:** 2025-06-10

**Authors:** Li Yan, Alberto J. Huertas-Alonso, Hai Liu, Lin Dai, Chuanling Si, Mika H. Sipponen

**Affiliations:** a State Key Laboratory of Bio-based Fiber Materials, Tianjin Key Laboratory of Pulp and Paper, College of Light Industry and Engineering, Tianjin University of Science and Technology Tianjin 300457 China dailin@tust.edu.cn sichli@tust.edu.cn; b Department of Chemistry, Stockholm University SE-10691 Stockholm Sweden mika.sipponen@su.se; c Wallenberg Wood Science Center, Department of Chemistry, Stockholm University SE-10691 Stockholm Sweden

## Abstract

Lignocellulosic biomass is the only sufficiently available resource for the sustainable development of the bioeconomy. Among the main components of lignocellulose, lignin has a tremendous potential to serve as a natural aromatic polymer resource due to the vast amounts of lignin available from industrial processes. However, commercial application of lignin is still limited and represents only a minor fraction of the potential utilization of approximately 20 million tons that can readily be isolated from spent pulping liquors and obtained as a residue from lignocellulosic biorefineries. Industrial processes generally depolymerize lignin into heterogeneous mixtures of low molecular weight macromolecules with a high degree of condensation, which collectively makes it challenging to develop them into high-performance materials. Although often neglected, some of the major limitations of these so-called technical lignins are their low molar mass and high dispersity, which make these lignins have poor mechanical properties. The polymerization of small lignin fragments not only contributes to the development of high-performance and multifunctional advanced materials, but also helps to improve the fundamental theory of lignin polymer chemistry. In this review, the polymerization of lignin *via* physical (aggregation), chemical (chain extension, cross-linking, and grafting), and biological (enzymatic polymerization) routes is described, its applications are assessed, and prospects for the development of high-performance lignin polymer materials are discussed.

## Abstract 2

Native lignin is fragmented during biorefinery processing into heterogeneous oligomers, limiting its material performance. Polymerization strategies hold promise for the design of high-performance lignin-based materials.

## Graphical abstract

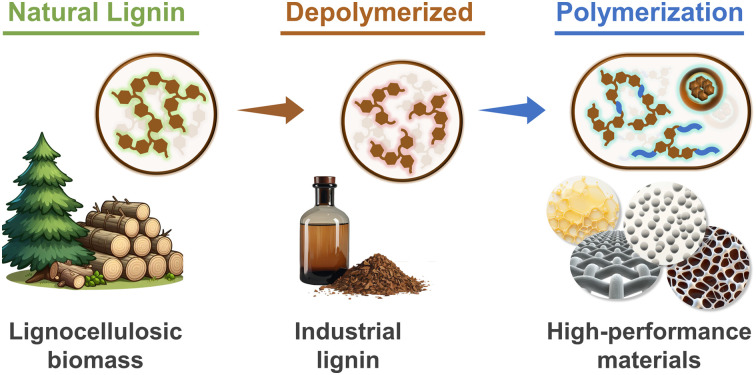

## 1. Introduction

Lignin is a polyphenolic component of wood found in the cells of nearly all land plants, wherein it provides structural support, facilitates long-distance water transport, and acts as a barrier against pathogens and microbes. Lignin is formed by the oxidative radical coupling and dimerization of phenolic monomers.^1^ The resulting lignin macromolecule contains functional group sites that can be modified, including methoxy, alcohol hydroxyl, phenolic hydroxyl, and carbonyl groups. The intricate three-dimensional architecture endows lignin with a variety of natural properties, including adhesion, hydrophobicity, antioxidant, and UV-shielding. In addition to its natural functions within the plant, the addition of artificially extracted lignin can enhance nutrient uptake in plants through ion exchange and complexation,^2^ while also improving soil porosity.^3,4^

The direct utilization of lignin as a substitute for petrochemical feedstocks in the production of polymeric materials represents one of the most straightforward routes for lignin valorization. However, in the current pulping and biorefinery processes, lignin undergoes depolymerization and condensation under harsh conditions. As a result, technical lignins typically exhibit a low molecular weight,^5,6^ a high degree of condensation,^6,7^ and high heterogeneity, which severely limit their utilization as material feedstocks, and thus they are still regarded as by-products to be separated, discarded, or burned for energy.^8,9^

Structural modification of lignin, particularly polymerization and supramolecular modification, represents an effective approach to enhancing the utilization value and expanding the application areas of technical lignins.^10,11^ For example, the chemical reactions possible with lignin—such as esterification, etherification, and amination—utilize its phenolic hydroxyl, aliphatic hydroxyl, and unsaturated groups, which enhance lignin's compatibility with nonpolar polymer matrices. Through grafting from and grafting onto copolymerization methods, lignin can be endowed with a wide range of functionality, and at the same time increasing the molecular weight and effectively improving the mechanical and thermodynamic properties of lignin-derived materials,^12^ which are of particular importance for membranes, coatings, adhesives, and other high-performance materials.^13,14^ Additionally, lignin molecules possess various unsaturated structures, including benzene rings, which facilitate a wide range of non-covalent interactions. These interactions lead to the formation of diverse structures, such as supramolecular particles, gels, and multiphase systems like emulsions.^15^ These materials exhibit adaptable and multifaceted surface chemistry and mechanical properties and show considerable promise in applications such as flame retardancy, food packaging, plant protection, and electroactivity.^16^ However, from the point of view of green chemistry, it would seem advisable to minimize the chemical derivatizations and maximize the bio-based content of the lignin polymers.

In light of the mounting environmental and resource depletion crises, there is a growing demand for more efficient and refined utilization of resources. Concurrently, the necessity for enhanced efficiency and control over the physicochemical modification of lignin has become increasingly apparent.^9^ In recent years, inspired by the natural lignification process, enzyme-catalyzed polymerization has emerged as a promising new method for lignin modification and polymerization,^17–19^ increasing the molar mass of birch alkaline lignin with 13.1-fold (*M*_w_ = 51 660 g mol^−1^).^20^ A patent application disclosed that an enzymatic treatment with bilirubin oxidase resulted in a 5-fold increase in the molecular weight of alkali lignin from Sigma-Aldrich, which was well-suited for carbon fiber manufacturing.^21^ Laccase-polymerized lignin can be employed to enhance lignin reactivity, augment hydrophobicity, and facilitate the synthesis of adhesives, coatings, thermosetting resins, carbon fiber precursors, agricultural fertilizers, deodorants, and other materials.^22^ However, challenges remain in understanding the competing mechanisms of polymerization and depolymerization as well as resulting noncovalent interactions between lignin and other molecules. The goal is to better control lignin polymerization and promote effective coupling reactions with target molecules.

**Fig. 1:**
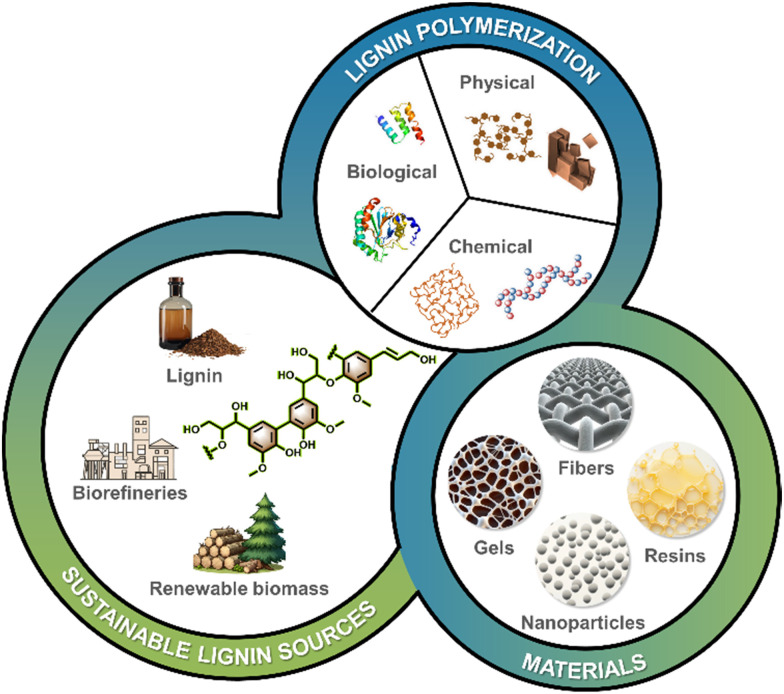
Overview of strategies for polymerization of lignin towards engineered materials.

This review provides a comprehensive outlook on strategies and applications of lignin-derived polymeric materials (resins, nanoparticles, fibers and gels, see Fig. 1). Several recent reviews on the topic of “lignin polymeric materials” are available, with the majority focusing on the lignin-derived chemicals, associated polymers and applications. Herein, we address the polymerization of lignins sourced from industry, with the objective of increasing their molecular weight and ability to control fabrication of supramolecular materials *via* aggregation, thereby enhancing their application value. In addition, the substantial potential for environmental protection, biomedicine, and other high-value applications of lignin-based polymeric materials has been elucidated. Moreover, the future trajectories and potential advancements of lignin-based polymeric materials have been analyzed to enable the fabrication of high-performance lignin-based materials with advanced functionalities. The principles put forward herein can be applied to the modification and functionalization of other polyphenols such as tannins and their mixtures with other biomass components.

## 2. Polymerization strategies for lignins

Based on controlled bottom-up synthesis at different molecular scales, lignin polymerization approaches can be categorized into physical, chemical, and biological methods. We critically analyze these polymerization routes, including physical aggregation, covalent cross-linking, chemical chain extension, polymer grafting, and enzyme-catalyzed polymerization reactions. We begin with an overview of the current understanding of lignin biosynthesis, focusing on its polymerization within plant tissues.

### 2.1. Biological polymerization of lignin

**Fig. 2:**
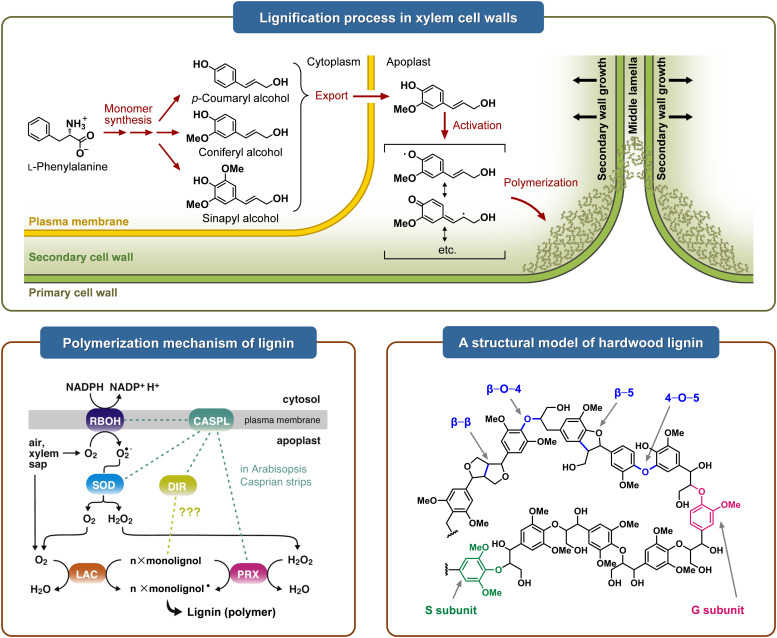
An overview of processes, adapted from ref. 25 with permission from Annual Reviews, copyright 2010, mechanisms, adapted from ref. 23 with permission from Elsevier, Ltd, copyright 2019, and structures relevant to lignification, adapted from ref. 26 with permission from Springer Nature, Ltd, copyright 2022.

Lignin, an intricate polyphenolic macromolecule, is typically interlaced with cellulose and hemicellulose to augment the mechanical robustness, rigidity, and hydrophobicity of plant secondary cell walls. Lignification occurs in a late phase of plant development, forming rigid woody tissues that resist digestion, even by ruminants.^23^ The oxidases and peroxidases in the cell wall, whether free or bound, determine the spatial localization of lignification by catalyzing the polymerization of lignin monomers (Fig. 2).^24^

Laccases, classified as *p*-diphenol dioxygen oxidoreductases (EC 1.10.3.2), are oxidoreductases that can initiate the polymerization of lignin and are widespread among bacteria, fungi, higher plants, and insects.^27^ Laccases can act *via* different mechanisms depending on the substrate and the presence of mediators.^28^ Laccases catalyze the oxidation of phenolic compounds by proton abstraction, generating phenoxy radicals, which can undergo further coupling.^29^ This reaction is facilitated by a multicopper active site that transfers electrons from the substrate to the molecular oxygen, reducing it to water. Laccase-catalyzed systems facilitate the oxidation of non-phenolic substrates through redox cycling of low-molecular-weight mediator compounds. Prominent mediators employed in these systems include 1-hydroxybenzotriazole, violuric acid, and 2,2′-azino-bis(3-ethylbenzothiazoline-6-sulfonic acid), which collectively enhance the oxidation efficiency of recalcitrant lignin structures.^30^ However, laccases may give rise to competing depolymerization and repolymerization reactions, as evidenced by the formation of both low-molecular-weight degradation products and high-molecular-weight oligomeric species.

**Fig. 3:**
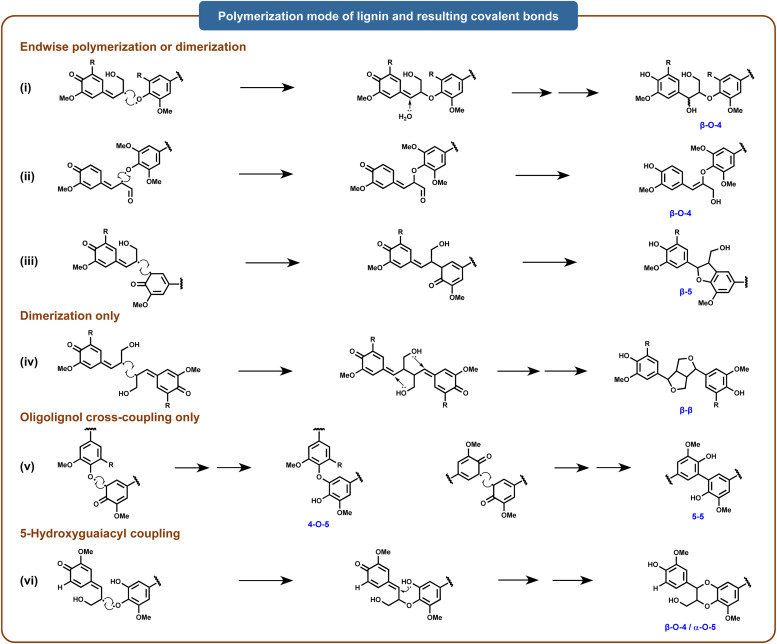
Radical coupling of major structural units in the lignin polymer. Adapted from ref. 25 with permission from Annual Reviews, copyright 2010.

The polymerization of lignin involves the terminal polymerization of monomers to oligomers, the coupling of oligomers, and the dimerization of monomers. The most significant linkages resulting from these reactions include β-O-4′, β-5, β-β′, 5-5′, and 4-O-5′.^31,32^ Specifically, laccases can directly catalyze the oxidation of phenolic compounds in organisms to reactive phenoxy radical intermediates, which can spontaneously couple *via* carbon–carbon, carbon–nitrogen, and carbon–oxygen (ether) bonds to form a diverse array of dimeric isomers.^33^ Moreover, as these dimers retain phenolic hydroxyl groups, they can be further oxidized by laccases to form additional reactive radical intermediates, which then undergo oxidative coupling with phenolic substrates to form structurally complex oligomers and polymers.^34^ Laccase-mediated electron transfer between phenoxy radical intermediates generates diverse polymerization products, expanding the variety of resulting polymers^23^ (Fig. 3). For instance, a bacterial-derived alkaliphilic laccase has been demonstrated to catalyze the oxidation and polymerization of isolated birch alkaline lignin fractions, resulting in a 13.1-fold increase in molar mass for the B-i-PrOH-s fraction after six hours of treatment.^20^ Studies suggest that laccases can promote lignin polymerization with various functional molecules, including phenolic derivatives (vanillic acid, gallic acid, tannic acid), nitrogenous compounds (isocyanates and N–OH-type mediators), acrylic monomers, chitosan, soy protein, amine and amide polymers, cellulose, and inorganic polymers.^35^

Peroxidases (PRXs) have also been demonstrated to play a role in the lignification process. It is thought that different PRXs are active at specific tissues, cell types, or developmental/growth stages.^36^ The mechanism underlying lignification catalyzed by PRXs bears resemblance to that of laccases, whereby free radicals are generated from substrates, which then undergo secondary reactions.^29,37^ The distinction between these two processes lies in the fact that laccases utilize oxygen as a co-substrate, whereas the oxidative cycle of peroxidases relies on hydrogen peroxide.^36^ Hydrogen peroxide can be produced within the cell wall by enzymes such as nicotinamide adenine dinucleotide phosphate (NADPH) oxidase, amine oxidase, and oxalate oxidase. Additionally, under certain conditions, PRXs themselves can also generate hydrogen peroxide.^24^ Prior research has indicated that distinct peroxidases display a tendency for oxidizing particular lignin monomers. A multitude of variables may impact the catalytic activity of PRXs and the configuration of the resulting lignin, including the biological medium, concentrations of lignin monomers and hydrogen peroxide, and the specific PRXs involved. Analogous to laccases, PRXs can also facilitate the graft polymerization of lignin with other functional small molecules.^38^ Moreover, enzymatic polymerization of lignin has been demonstrated to correlate with the molecular weight of the lignin substrate, with low molecular weight substrates generally more reactive than the larger macromolecules.^17,39,40^

It is becoming clear that the enzymatic process coupling phenolic compounds to form polymerized lignin is highly complex and goes beyond the traditional models involving laccases and peroxidases.^41^ A recent study identified a class of dirigent domain-containing proteins (DIRs) that modulate the primary structure of lignin in plant roots by controlling the regio/stereochemistry of free radical coupling reactions (Fig. 2). By examining the expression patterns of 25 DIRs in Arabidopsis, Gao discovered that five genes (DIR9, DIR24, DIR16, DIR18, and ESB1) exhibited specific expression in the root endodermis. The localization of all selected DIRs in the endodermis, particularly in the Casparian strip, was confirmed by fusion with the fluorescent protein mCherry. In single DIR mutants, the integrity of the Casparian strips was assessed by detecting lignin deposition at the strips using basic magenta staining. Furthermore, the researchers explored the impact of environmental factors on DIR activity within plants. This work revealed a Casparian strip lignification mechanism that necessitates collaboration between DIR and the Schengen pathway.^42^ The investigation was extended to the origin of enzymes, with both bacteria and fungi demonstrating the potential for the polymerization of lignin-related small molecules.^43^

### 2.2. Physical aggregation of lignin

**Fig. 4:**
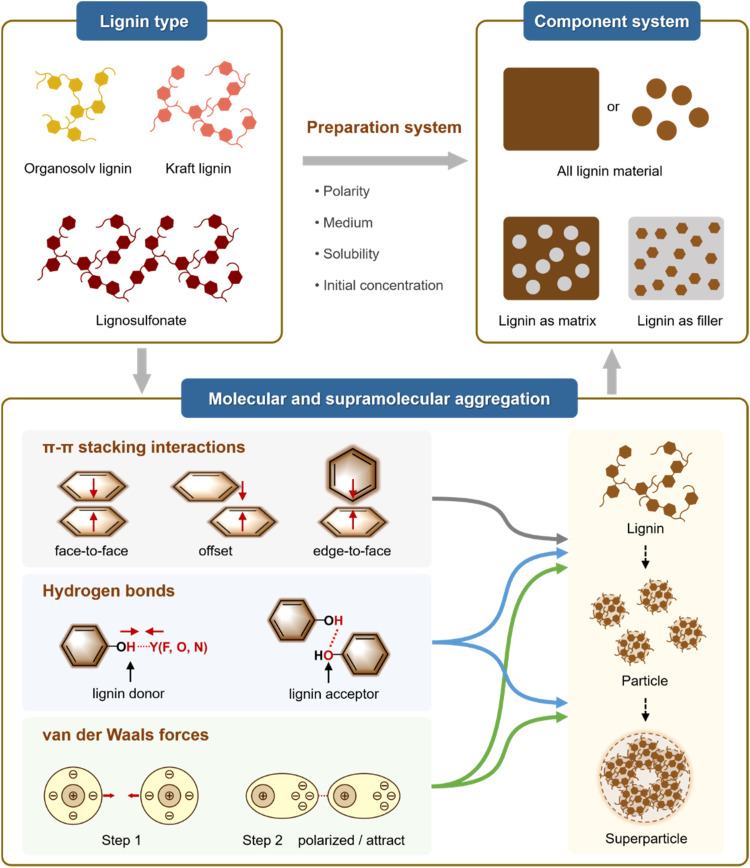
Physical aggregation of lignin *via* attractive intermolecular forces.

Physical aggregation of lignin is facilitated by a variety of intermolecular and intramolecular interactions, which may act in a repulsive or cohesive manner, including electrostatic interactions, hydrogen bonding, π–π stacking interactions, and van der Waals forces (Fig. 4). The amphiphilic nature of lignin also promotes the formation of aggregates that reduce surface energy in a given solvent. Lignin inherently possesses a multitude of hydrogen bond donor and acceptor groups, including hydroxyl, carboxyl, and ether groups.^44^ This chemical structure results in the prevalence of hydrogen bonds within and between lignin molecules (*e.g.*, β-O-4′)^45–47^ and also between lignin and other substances, including solvents,^48,49^ significantly impacting the solution behavior of lignin.^50^ Hydrogen bonding is considered a key driving force for the aggregation behavior of lignin in solvents and can be manipulated through chemical modification of its functional groups.^51^ Additionally, at certain solid interfaces, such as inorganic oxides, hydrogen bonding plays a crucial role in the adsorption and dispersion behavior of lignin-based dispersants.^52^

Attractive forces resulting from π–π interactions between the benzene rings of proximal phenylpropane units play a pivotal role in lignin aggregation.^53,54^ This was supported by density functional theory (DFT) modelling of representative structural motifs.^55^ Based on theoretical calculations and experimental verification, Hunter *et al*. attributed π–π stacking to the attraction between disparate electron clouds between aromatic systems.^56^ The interaction energies depend on the relative positions of the two aromatic rings, which can include face-to-face stacking, offset stacking, and edge-to-face stacking.^50^ Through computational quantum chemistry methods, including counterpoise-corrected aug-cc-PVTZ basis sets and MP2-R12/A computations, Sherrill *et al*.^57^ demonstrated that offset stacking and edge-to-face stacking represent the most stable configurations in the simplest model of aromatic π–π interactions, the benzene dimer, which correspond to energy minima. Conversely, face-to-face stacking is the least stable configuration and represents an energetic saddle point, because this kind of configuration maximizes the overlap of the π electron systems.^58^ Xiang *et al.* explored the relationship between the conjugation of lignin and its supramolecular structure and also demonstrated that the ordered and compact supramolecular structure results in a shorter average distance between benzene rings and stronger π–π interaction, which leads to altered properties, *e.g.*, increased ultraviolet-shielding property, photothermal conversion capability and the adsorption behavior.^54,59^ Noteworthy, the three-dimensional branching of lignin presents steric hindrance, and not all the aromatic groups, but only the aromatic moieties with zero net charge can form π–π stacking.

Lignin exhibits amphiphilic properties and can form aggregates with hydrophobic cores when precipitated in a controlled manner from a suitable solvent system. Technical lignins, which are often modified, contain ionizable functional groups such as carboxylic acid and sulfonic acid groups, which significantly influence the behavior of lignin through electrostatic interactions.^60,61^ Water plays a pivotal role in the manifestation of hydrophobic interactions and electrostatic forces,^62^ which are responsible for the repulsion between like charges and the attraction between opposite charges.^63^ For example, under alkaline conditions, the deprotonation of acidic groups in kraft lignin induces substantial surface charge development, thereby enhancing colloidal stability through electrostatic repulsion mechanisms. Conversely, acidification promotes protonation of these groups, effectively neutralizing surface charges and initiating molecular aggregation through hydrophobic interactions and hydrogen bonding to form stable particles or precipitates.^64^ This colloidal system exhibits concentration-dependent stability, with a direct correlation to both the absolute surface charge density and its spatial distribution across the lignin macromolecules.^65^ When the lignin concentration exceeds a critical value, bridges can begin to form between these colloidal particles, resulting in the formation of a networked structure.^66^ Once aggregated and due to the expulsion of water from the particle interior the proximal lignin molecules are additionally stabilized by van der Waals forces that originate from the transient dipole moments of atoms or molecules, resulting in an attractive force at short separation distances, which is composed of orientation, induction, and dispersion forces. For lignin with a comparable structure and composition, a higher molecular weight results in the exertion of stronger aggregate van der Waals forces.^67^ Compared to hydrogen bonding, van der Waals forces have a similar or slightly stronger magnitude.^68^

Aggregation phenomena are also central in lignin-related material systems that can be classified into three categories: all-lignin materials, lignin matrices, and lignin fillers. All-lignin materials are emerging as promising candidates in fields where recyclability and low toxicity are desired.^69^ When employed as a matrix, lignins are frequently combined with other functional materials to impart novel properties to the resulting material. As a reinforcing filler, lignin may enhance mechanical properties, depending on the weight fraction.^70–74^ In most cases, increasing the lignin content of the composite above 10–20% leads to deteriorated mechanical properties.^75–77^ To enhance interfacial compatibility with other polymers, chemical modifications are typically required. For example, using lignin as the raw material of polyurethane, combining lignin with the Diels–Alder (DA) reaction markedly elevates the tensile strength of lignin-polyurethane composites.^78^ Beyond that, esterification of lignin augments the flexibility of lignin-polypropylene complexes, and poly(methyl methacrylate)-grafted lignin boosts both tensile strength and elongation at break.^79^

### 2.3. Chemical polymerization of lignin

**Fig. 5:**
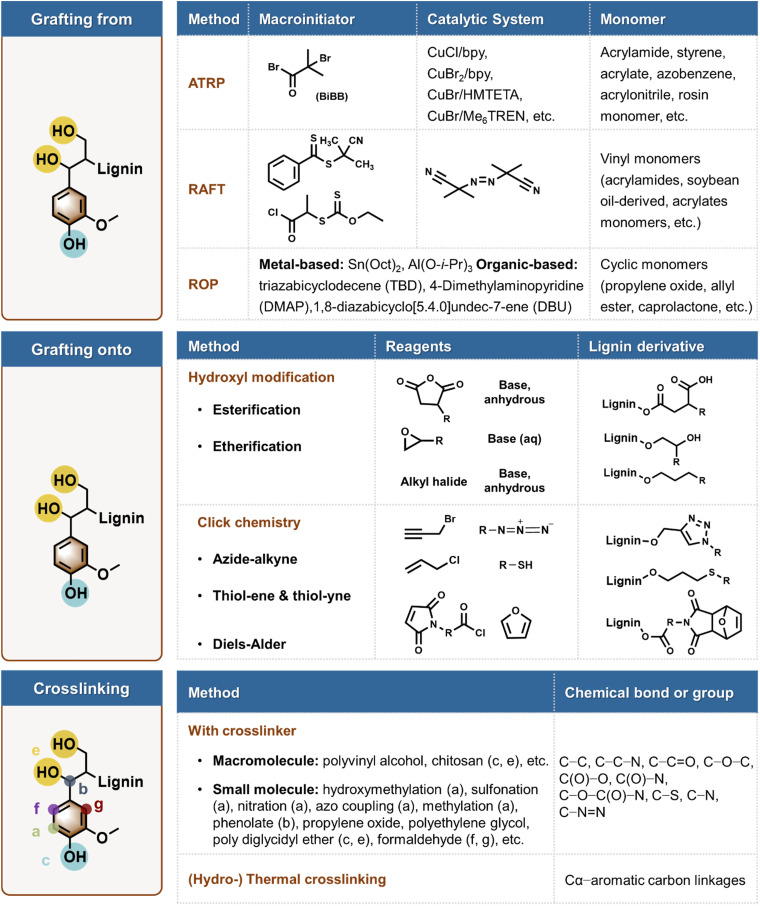
Examples of common routes for chemical polymerization of lignin *via* grafting from, grafting onto, and crosslinking chemistries.

Chemical polymerization of lignin compasses polymer grafting and crosslinking chemistries summarized in Fig. 5. Below, we give the central features of these processes and their pros and cons from the point of view of green chemistry.

#### 2.3.1. Lignin-based co-polymers

Among various polymerization strategies of lignin, graft copolymerization has the potential to result in products with well-defined properties, which can be determined by the functional groups on the grafted polymers, the length of the graft, and graft density. There are two primary grafting methods: grafting from and grafting onto.^79^ In the grafting from method, lignin usually functions as the backbone polymer, with grafted polymers being synthesized from initiating sites on lignin by various polymerization methods, including atom transfer radical polymerization (ATRP), reversible addition-fragmentation chain transfer polymerization (RAFT), and ring-opening polymerization (ROP) (Fig. 5).^80,81^ The grafting onto process of lignin typically involves free radical polymerization, whereby certain monomers are activated under the influence of light, heat, radiation, or initiators, subsequently engaging the formed free radicals in polymerization with other monomers.^82,83^ Lignin copolymers can be synthesized *via* ATRP by establishing a reversible dynamic equilibrium between active and dormant species using simple organic halides to synthesize the lignin macroinitiators and transition metal complexes as halogen atom carriers.^84^ In the RAFT reaction involving lignin, dithioester derivatives are typically employed as chain transfer agents, which form dormant intermediates with growing chain radicals, thereby limiting irreversible termination side reactions and effectively controlling the polymerization. The common reactive monomers include acrylamides, acrylates, and soybean oil-derived epoxies.^85^ In the ROP process, the phenolic hydroxyl group of lignin serves as a reactive center where cyclic monomers can react *via* ring opening to form a longer polymer chain, facilitated by metal- and organic-catalysts.^86,87^ For example, graft polymerization of lactide to lignin catalyzed by 4-dimethylaminopyridine (DMAP) resulted in a lignin-*g*-poly(lactic acid) copolymer, which chain length can be controlled by varying the lignin/lactide ratio.^88,89^ Li *et al*. reported that ROP has occurred with either aliphatic or phenolic hydroxyls, but there was a preference for aliphatic ones in both cases.^90^

Grafting onto requires efficient covalent bond forming reactions between the lignin backbone and the end groups of the pre-synthesized polymers. The grafting onto process involves the derivatization of lignin with functional groups, which serve as sites for attaching *ex situ* synthesized polymer chains to produce the final grafted polymer.^11^ This approach contrasts with grafting from in that it allows for the incorporation of a more diverse range of polymers, offering more convenient reaction conditions, and providing a broader selection of solvents and purification methods. One strategy for attaching a separately synthesized polymer onto lignin is through atom-efficient click reactions.

Click chemistry is recognized for its convenience and efficiency, characterized by stereo- and regio-specific mechanisms that lead to controlled chemical structures.^91^ It typically involves minimal side reactions, yields a singular product, and can be performed at ambient temperature and pressure in “green” solvents or bulk.^92^ Lignin's reactive hydroxyl groups are particularly well-suited for derivatization for “click” reactions. Among these, lignin is chemically modified with alkyne to participate in the Huisgen 1,3-dipolar cycloaddition of alkynes and azides to form 1,2,3-triazoles.^93^ In addition, the copper-catalyzed azide–alkyne cycloaddition (CuAAC) reaction (a variant of the classical Huisgen 1,3-dipolar cycloaddition, developed by the groups of M. Meldal and K. B. Sharpless) is commonly employed due to its high efficiency and the convenience it offers in conducting the reaction.^94,95^ The CuAAC reaction offers a 100-fold increase in rate over non-catalytic 1,3-dipole cycloaddition reactions. It operates over a wide range of temperatures, is insensitive to water, functions in the pH range of 4 to 12, and is compatible with many functional groups. Pure products can be obtained by simple filtration or solvent extraction, obviating the need for column chromatography or recrystallisation.^96^ Reactions between thiols and enes (radical thiol-ene or anionic/nucleophilic Michael addition) exhibit numerous characteristics associated with click reactions. Lignin monomers are capable of undergoing radical thiol-ene reactions with suitable alkene moieties, provided that the phenolic groups are either protected or derivatized.^97^ However, literature is lacking demonstration of such direct reactions with conjugated alkenes with macromolecular lignins.

The furan-maleimide Diels–Alder reaction has been extensively utilized for its relatively mild conditions, which enable an on-demand depolymerization–repolymerization process.^98^ Maleimide groups can be introduced onto lignin, which subsequently undergoes a thermally reversible click reaction with additional maleimide groups.^99^ These moieties are of particular interest for the design of novel macromolecular structures of lignin. For example, 6-maleimide hexanoic and 11-maleimide undecanoic acids (11-MUA) can be converted into their corresponding acyl chlorides and then grafted onto lignin by esterification of both phenolic and aliphatic hydroxyl groups, resulting in a conversion rate of up to 90% of the total OH groups.^100^ The grafting of alkyne-terminated carboxylic acids, such as 5-hexynoic acid onto phenolic and aliphatic hydroxyl groups of lignin *via* carbodiimide-mediated esterification has been demonstrated to achieve alkyne functionalization.^101^ To date, there have been few studies exploring the thiol-yne polymerization of lignin or other biobased polyphenols.^102^ In addition to functionalization, these reactions can also help increase the molecular weight of lignin.^103^ Another type of atom-efficient “click” reaction, which does not necessitate the functionalization of lignin, is the reaction between an allyl group and the phenolic hydroxyl group of lignin. This chemistry results in the formation of dynamic acetal linkages, which can be employed to synthesize lignin-based vitrimers, a class of recyclable thermosets.^104^

#### 2.3.2. Chain extension and cross-linking chemistry

The low molecular weight of lignin is a major shortcoming in polymeric materials. Covalent chemical polymerization routes are the most prevalent approaches to increasing the molecular weight of lignin, encompassing chemical grafting and cross-linking techniques.^23,105^ In addition to the reactive aromatic ring positions, the phenolic and aliphatic hydroxyl groups within the lignin structure provide reactive sites for chemical polymerization (Fig. 5). One example of such cross-linking approaches targeted the aromatic ring carbons through hydroxymethylation using formaldehyde, followed by a condensation reaction that formed methylene bridges. This approach increased the molecular weight of a hardwood kraft lignin from 1840 g mol^−1^ to 31 000 g mol^−1^.^106^

**Fig. 6:**
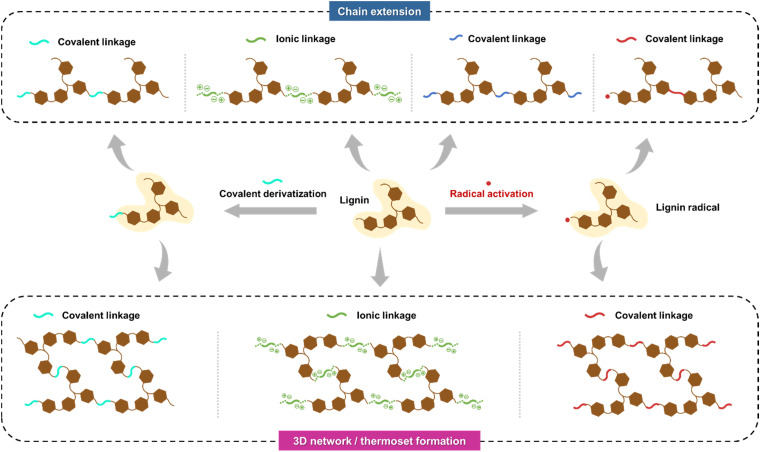
Illustration of competing chain extension and 3D network/thermoset formation from lignin.

A prevailing challenge in lignin cross-linking chemistry is achieving polymer chain extension rather than forming a three-dimensional polymer network (Fig. 6). Due to lignin's inherent multifunctionality and branched structure, the resulting polymeric matrices often form thermosetting networks with poor processability. Control over chain extension has been at least partially achieved through propargylation and subsequent methylation of the acetone-soluble fractions of softwood kraft lignin.^107,108^ After selective methylation, the benzopyrans, arising from the Claisen rearrangement between the propargyl groups and the free C5 positions of the propargylated lignin, can be thermally polymerized to high molecular weights in a controlled manner. Such selective derivatization and polymerization reactions present attractive directions for further research that could bridge gaps between structure–property–performance relationships and aim to achieve favorable green metrics, such as a low E-factor, for the entire process.

The aromatic ring modification has also been achieved *via* thermal treatment at 148 °C (exceeding its glass transition temperature) of softwood kraft lignin, which increases molecular weight, likely due to the radical formation and the creation of carbon–carbon (5-5′) and ether (4-O-5′) linkages.^109^ Moreover, recent studies have indicated that hydrothermal treatment can cleave the Cα–Cβ and β-O-4′ ether interunit linkages, methoxy, and hydroxyl groups in lignin,^110^ leading to the generation of free radicals and the subsequent formation of more stable cross-linking structures.^111,112^ Many other chemistries targeting the hydroxyl functional groups of lignin have been extensively studied in recent years. Examples include the use of difunctional cross-linkers, such as sebacoyl chloride, to form polyester networks,^113^ or thiol-ene cross-couplings with allylated lignin.^114,115^

## 3. Applications of polymeric lignins

**Fig. 7:**
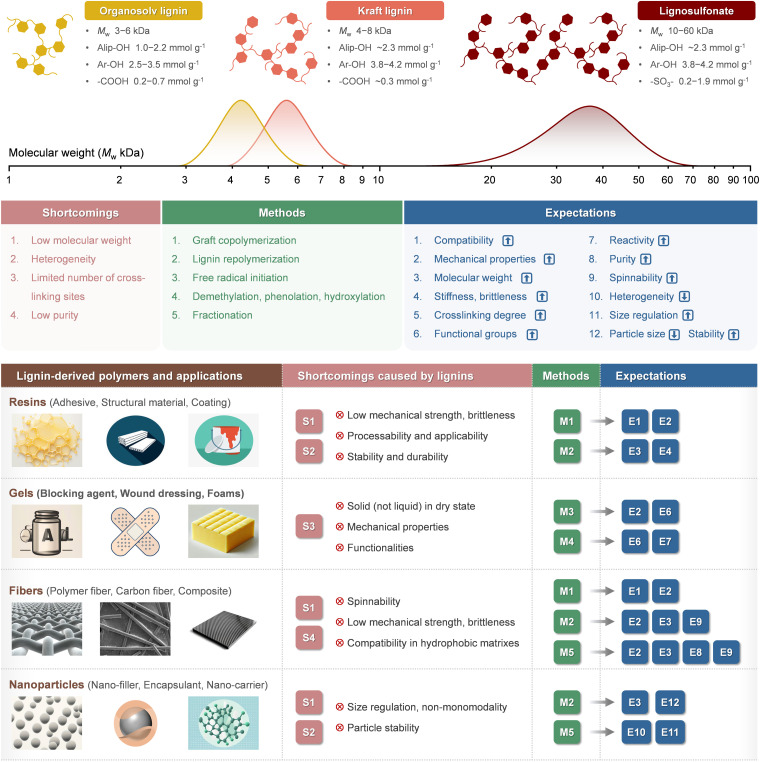
Properties of common technical lignin grades, shortcomings and suggestions for improvement towards applications of lignin-based polymeric materials.

The rapid advancement of lignin chemistry and analytical techniques has led to a growing understanding of the molecular structure of lignin, which has in turn driven the development of chemical modification methods. Lignin molecular modification technologies have facilitated the creation of multifunctional lignin-based composites, fibers, and micro/nano materials. Structurally, polymeric lignins exhibit longer molecular chains, higher molecular weights, and a higher degree of molecular branching (Fig. 7). In addition to the inherent properties of lignin itself (biocompatibility, biodegradability, recyclability, antibacterial and UV resistance, adhesive properties, *etc.*), polymerization will bring about performance enhancements resulting from improved mechanical properties, thermal stability, spinnability, electrical conductivity, adsorption, *etc.* These properties will find widespread application in the fields of biomedical, electronic, environmental, and energy (Fig. 7).

Lignin is commercially utilized as a natural adhesive or binder in phenolic resins for applications such as particleboard, plywood, and animal feed pellets. It is also employed as an additive or filler in plastics and rubber to enhance UV resistance and thermal stability and to reduce reliance on fossil-derived components.^116,117^ Although the correlation between lignin's molecular weight and its performance in these contexts has not been conclusively established, it is plausible that higher molecular weight fractions may offer improved mechanical properties, provided that miscibility with the polymer matrix and adequate reactivity or stability are maintained.

### 3.1. Resins

Lignin-based polymers can be classified into three main categories, thermosetting resins (epoxy resins, phenolic resins, and polyurethanes), thermoplastics, and vitrimers.^118^ These categories represent a spectrum of performance characteristics, with vitrimers exhibiting properties between thermosetting and thermoplastic materials. Epoxy resins, a representative thermosetting polymer material, are a prominent example where lignin has been employed as a substitute for bisphenol A and utilized in the reaction with ethylene oxide and polyether amine.^119^ The incorporation of lignins with higher molecular weights contributes to the enhancement of the epoxy cross-linking network, resulting in increased rigidity. The incorporation of lignin into epoxy thermosets results in an increase in structural flexibility, which is attributed to the ether linkages present in lignin.^120^ Another example is the modification of lignosulfonate by methacryloyl chloride and epichlorohydrin. The introduction of C=C enables copolymerization within the epoxy system, forming a dual interpenetrating network under the influence of an initiator. The resulting lignin-based adhesive exhibits notable tensile shear strength (11.3 MPa) and water resistance (9.3 MPa after 12 h in boiling water).^121^ A multitude of studies have prepared lignin-based epoxy pre-polymers directly through epichlorohydrin modification. After blending with bisphenol A diglycidyl ether (DGEBA), these lignin-based epoxy thermosets exhibit enhanced mechanical and thermal properties. Moreover, acetylated lignin exhibits an absorption capacity across the entire range of UV radiation,^122^ which may be harnessed as UV-protective coatings. However, it is important to note that UV absorption can cause oxidative degradation of lignin under environmental conditions.^123^

Polyols and phenols utilized in the production of polyurethane and phenolic resins can be partially or fully substituted with lignin and its derivatives, resulting in materials with enhanced mechanical strength and thermal stability.^124^ These improvements typically correspond to lignin incorporation ratios of up to 50%.^125^ The synthesis of lignin-based polyurethane materials by de Oliveira involved the reaction between lignin's hydroxyl groups and isocyanate, resulting in materials with superior impact resistance and modulus of elasticity compared to hydroxyl-rich castor oil polyurethanes.^126^ Outstanding challenges of the lignin-based polyurethanes include moving to non-isocyanate chemistries and solvent-free systems. In the case of lignin-modified phenolic resin, Çetin and Özmen synthesized phenolated-lignin formaldehyde resins with up to 30% lignin substitution rates, viscosity of 200–250 cP, and a cure temperature of 150 °C.^127^ Yang *et al.* reported a two-step strategy to produce lignin-formaldehyde adhesive by acid-mediated methylolation of aromatic C2/6 positions, resulting in a lighter color and far better adhesion performance (1.33 ± 0.08 MPa) compared to the minimum industrial requirement of 0.70 MPa.^128^

Lignin-containing composite materials can be obtained through the melt or solution mixing of lignin and thermoplastic polymers, accompanied by hydroxyl group blocking reactions.^129^ The common thermoplastic polymers including polyethylene, polypropylene, polyvinyl chloride, polymethyl methacrylate, polyvinyl alcohol, ethylene vinyl acetate copolymer, polyester, acrylonitrile butadiene styrene plastic, starch, and protein.^82^ For example, acrylonitrile butadiene styrene (ABS) has been utilized as a thermoplastic polymer matrix for lignin due to its favorable fused-deposition modeling (FDM) compatibility, mechanical performance, and melt properties.^130^ Nguyen *et al*. introduced a pioneering class of high-performance printable lignin-modified nylon composites, which contain 40 to 60 wt% lignin. When the addition of 40 wt% hardwood in nylon 12, the tensile Young's modulus increased from 1.77 ± 0.15 GPa to 3.01 ± 0.59 GPa. The presence of lignin and carbon fibers impedes the crystallization of nylon, resulting in the formation of imperfect crystals that enable good printability at 170 °C without the degradation of lignin.^131^ Mimini *et al*. reported that organosolv hardwood lignin exhibits good compatibility with PLA, endowing the blend with good printability by FDM at a 15 wt% lignin content.^132^

Many thermosets exhibit exceptional thermal, mechanical, and dimensional stability due to insoluble and infusible cross-linked networks, but are limited in their ability to be processed and reshaped.^133–135^ Thermoplastics are highly processable but have poor solvent resistance.^136^ Vitrimers have been proposed as a potential solution, consisting of a highly cross-linked covalent adaptive network (CAN) with reversible chemical bonds.^137,138^ This approach often combines the stiffness and chemical resistance of thermosets with the good processability and recyclability of thermoplastics.^139^ Furthermore, incorporating lignin as a feedstock reduces the environmental and resource impact of this class of polymers.

Zhang *et al*. have developed a lignin-based vitrimer through the reaction of ozonated lignin with sebaceous acid-derived materials in the presence of a 10 mol% zinc acetoacetonate, which undergoes bond exchange under thermal stress. The tensile strength, modulus, and glass transition temperature of these materials demonstrate an increase in value with an increase in the ratio of lignin loading. The application of external pressure results in deformation of the sample, whereas transesterification reactions facilitate the restoration of the cross-linked structure.^140^ Moreno and co-workers successfully engineered a series of lignin-based vitrimers based on dynamic acetal covalent networks, through a one-pot, thermally activated, catalyst-free “click” addition reaction of softwood kraft lignin (SKL) with poly(ethylene glycol) divinyl ether (PDV). The mechanical attributes of the vitrimer can be readily adjusted.^104^ Similarly, the mechanical properties can be modified by varying the ratio of hard to soft segments through imine chemistry.^141^ By means of a non-catalytic and cross-linked reaction, Liu *et al.* prepared a vitrimer material that is fully biobased, using wheat straw lignin. At elevated temperatures (>100 °C), dynamic chemical bonds inside the material are activated (*i.e.*, vinylogous urethane amine exchange), which allows it to be recycled and reused.^142,143^

It is worth noting that the majority of the reported vitrimers have been activated by heat treatment, which in turn enables secondary processing by activating CANs. This approach may, however, present a limitation in the thermal stability of the material, thereby narrowing the scope of potential applications.^144,145^ However, on the bright side, this limitation can be addressed by activation by a variety of external stimuli other than heat, including light, electricity, and chemistry.^146^ The advancement of photothermal conversion, pH response, and other lignin-specific functional properties, along with more flexible and controllable vitrimer chemistries, will infuse new vigor into the processing and application of novel polymer materials.

### 3.2. Fibers

The common preparation methods for lignin fibers include melt spinning, solution spinning, and electrospinning.^147^ The diameter, morphology, and mechanical properties of the resulting fiber materials are intimately connected to the chosen preparation method.^148^ The molecular weight and chemistry of lignin directly impact its spinnability and the mechanical properties of the derived fibers. Consequently, it is crucial to develop techniques for the synthesis of polymeric lignin with suitable chemical functional groups.

Without chemical modification lignins typically possess insufficient melt-processability. The production of lignin-based polymeric fibers typically entails the incorporation of lignin into a host polymer matrix.^3^ Polymers frequently employed for this purpose include polyethylene oxide (PEO), thermoplastic polyurethane (TPU), polyethylene terephthalate (PET), polylactic acid (PLA), among others. The compatibility and interaction between the lignin and the host polymer play a pivotal role in determining the mechanical properties of the resulting fibers. For instance, TPU containing hydroxypropyl-modified lignin exhibits enhanced spin-softening performance due to favorable compatibility and hydrogen bonding interactions.^149^ Polyesters such as PET and PLA, which possess inherent melt-spinnability, can also strongly interact with lignin molecules, making them popular choices for the creation of lignin-based fibers.^150^

Lignin is regarded as a possible substitute for polyacrylonitrile (PAN) and other petroleum-based polymers in the production of carbon fibers (CFs) due to its low cost, renewability, a high proportion of aromatic hydrocarbon benzene rings, and elevated carbon content ranging from 60% to 66%.^151,152^ The manufacturing technology of lignin-derived CFs and their potential applications have been extensively investigated in a number of fields, including composite materials, energy storage, catalysis, ablative materials, and smart sensing technologies.^147,153–155^ For example, the incorporation of PEO into lignin effectively enhances the softening ability of lignin during spinning, with carbonized fibers achieving tensile strengths and moduli of 400–500 MPa and 30–60 GPa, respectively.^153^ Jin *et al*. fractionated kraft lignin using an acetic acid and water solvent system, resulting in the isolation of three fractionated-solvated lignin precursors (FSLPs) with varying molecular weights (7.2, 13.8, and 28.6 kDa). Subsequently, lignin-derived CFs were produced *via* melt spinning and spinning the precursor filaments. The study demonstrated that the highest molecular weight lignin resulted in carbon fibers with the optimal mechanical properties, exhibiting a strength of 1.39 GPa and a modulus of 98 GPa.^156^ Additionally, lignin with a higher molecular weight and improved homogeneity exhibit better thermal stability. Li *et al*. investigated the correlation between the mechanical properties of lignin carbon fibers and the molecular structure of lignin using a linear regression model. Their findings indicated that carbon fibers with enhanced mechanical properties and higher electrical conductivity could be produced from lignin rich in linear β-O-4 bonds.^157^ Although lignin holds great promise for utilization within the CFs industry, at present, over 90% of commercial CFs are derived from PAN and pitch, which possess stable raw material properties, well-designed molecular structure and controllable spinning processes. Meanwhile, as the lignin fractionation technology and lignin polymerization strategies continue to evolve, the issues of inferior molecular weight and purity of lignin feedstock, moldability and subpar performance of lignin-derived CFs are undergoing enhancement.^158^

### 3.3. Gels

Gels are a viscoelastic material defined by a network of cross-linked molecules or particles that spans the entire system, immobilizing the liquid phase and conferring solid-like properties once a critical connectivity threshold is reached. Despite this structural complexity, the material appears macroscopically homogeneous, effectively averaging out the molecular-scale heterogeneity of lignins. This rationale also underlies the use of supramolecular assemblies such as lignin nanoparticles, which extend the relevant length scale and simplify the treatment of the material. The two most common gel materials are hydrogels and derived aerogels.^159^ The synthesis of lignin-based hydrogels is facilitated through various techniques, including cross-linking, copolymerization, and grafting.^160^ Lignin serves not only as a green feedstock for gels, but also improving their mechanical properties, and infusing them with functionality such as enhanced adhesion, conductivity, UV resistance, and antibacterial characteristics.^161^ In recent years, the applications of lignin gels have been reported across diverse fields, including biomedical, agricultural, environmental, and electronic sectors.

The incorporation of lignin with other polymers, including polyvinyl alcohol (PVA), PLA, acrylamide, chitosan, and cellulose, can markedly enhance the mechanical robustness, stability, viscoelasticity, ability to form a continuous and stable film, and water-absorptive capabilities of the resulting polymeric hydrogels.^162^ Furthermore, embedded polymers or oils can impart controlled-release properties to these materials. Such characteristics render lignin hydrogels particularly attractive for uses in daily care, tissue engineering, wound healing, drug delivery systems, and 3D bioprinting.^163^ Sipponen *et al*. report triglyceride oil-in-water emulsions stabilized in physical lignin gels, which can closely match a commercial hair conditioner product's rheological properties and hair conditioning action. This organic solvent-free and fully biobased hair conditioner simplifies the ingredient list and offers an environmentally benign route for lignin utilization in hair care.^164^ In another approach, Almenara *et al.* developed a fabrication process enabling the production of free-standing sodium lignosulfonate–chitosan and micellar lignosulfonate–kraft lignin–chitosan gel polymer electrolytes (GPEs) with diameters exceeding 80 mm. These GPEs afforded a more stable and homogeneous Zn electrodeposition on the electrodes during Zn stripping and plating.^165^ Dai *et al*. reported a pH-stimuli responsive lignin-based hydrogel by a one-step crosslinking reaction of industrial kraft lignin with poly(ethylene glycol)diglycidyl ether acting as a crosslinker. The resulting lignin-based hydrogel showed relatively fast (1 min) mechanical actuation by transition between softening/enhancement and straight/bending shapes by alternately immersing the hydrogel in acid and alkaline environments. Additionally, an intelligent hook and a flow control valve based on this system were demonstrated. This work provides a good example for the advanced applications of lignin-based hydrogels.^69^

Lignin-based aerogels can be fabricated through a sol-gel process that involves transforming lignin into a sol, followed by gelation and drying to yield the aerogel.^166^ The material produced by this method has been shown to comprise mainly mesopores and 10.5–28.9 nm in lignocellulose aerogels, the density of the bacterial cellulose-lignin resorcinol formaldehyde (BC-LRF16, lignin content 17 wt%) carbon aerogel increases to 0.026 g cm^−3^ from 0.013 g cm^−3^ of the pure BC carbon aerogel. These densities compare favorably to that of the carbon aerogel made from watermelon (0.058 g cm^−3^) and exhibit a *C*_total_ and a *C*_meso_ of 62.2 and 78.7 μF cm^−2^, respectively, much higher than *C*_total_ of titanium carbide-derived carbon (13 μF cm^−2^)^167^ and graphene (19 μF cm^−2^),^168^ which possess large total surface areas (1000 and 1310 m^2^ g^−1^, respectively). This property makes them suitable candidates for flexible solid-state energy storage devices. Besides energy storage, the conductive interconnected nanoporous structure can also find applications in oil/water separation, catalyst supports, sensors, and so forth.^169^ Cao *et al.* designed lignin-based multi-scale cellular aerogels assembled from co-electrospun nanofibers. The resulting lignin-based aerogel exhibits excellent hydrophobicity and high adsorptive capacity for diverse oils and organic solvents (adsorption capacity up to 103 g g^−1^). Moreover, the presence of nitrogen-containing functional groups enabled carbonization of the aerogel to nitrogen-doped carbon aerogels with the potential to serve as electrode material in supercapacitors. Its intricate micro- and nanoscale structures facilitate the rapid transportation of ions and electrons. The symmetrical supercapacitor assembled from it exhibits a high energy density of 32 W h kg^−1^.^170^

Looking ahead, the in-depth exploration of the inherent functional properties of lignin, such as pH-responsiveness, photothermal conversion capability, and UV resistance, position it as a promising component in gel materials. The three-dimensional macromolecular structure of lignin not only provides stable structural support, but its abundant functional groups also confer dynamic responsiveness to gels, enabling further development of advanced functional materials.

### 3.4. Nanoparticles

Lignin can be transformed into uniform nanoparticles through a variety of processes, including supramolecular aggregation of lignin *via* solvent exchange or acid precipitation.^171^ These particles can be broadly classified into three categories: pure lignin particles, lignin-encapsulated particles, and lignin blend particles.^172^ In recent years, the study of lignin nanoparticles has advanced rapidly and has been broadly applied in various emerging fields, including catalysis, CO_2_ capture, biobased adhesives, electrical devices, template materials, optical materials, and others.

Wang *et al*. proposed a strategy to convert disordered lignin into structured “photonic lignin”, which displays structural colors that can be adjusted by altering the diameter of lignin colloidal spheres. This photonic lignin exhibits vivid, angle-independent, and responsive structural colors.^173^ The layers of lignin nanoparticles are assembled in a manner that results in semi-closed packing structures, leading to coherent scattering. Liu *et al*. extended the centrifugation-assisted fabrication of photonic crystals that emit rainbow structural colors covering the entire visible spectrum. Their findings indicate that centrifugal forces are crucial for the formation of lignin photonic crystals, as the assembly of lignin nanoparticles without centrifugation leads to stripe patterns instead of photonic crystals. Additionally, centrifugation serves to classify lignin nanoparticles by size and produce monodispersed particle layers that display a gradient of colors from red to violet.^174^ A recent study introduced a high-yield method for fabricating photonic glass materials with no long-range order.^175^ By directly using crude lignin powders to prepare LNPs in ethanol and employing water as a poor solvent, tunable colors from blue to red were achieved with yields ranging from 48% to 72%. Elsewhere, a green and straightforward encapsulation method using industrial lignin has been reported for stable, uniform, and reproducible patterning of eutectic gallium–indium. This system enables fast and efficient recycling and rapid regeneration, making it highly environmentally friendly throughout the preparation process and finding applications in flexible sensors, transient circuits, and many other areas.^176^

Bisphenol A diglycidyl ether (BADGE), an aromatic cross-linker predominantly used in epoxy resins, was coprecipitated with softwood kraft lignin to form hybrid lignin-based nanoparticles (hy-LNPs). Hy-LNPs with BADGE content of at least 30% permit both inter- and intraparticle cross-linking at temperatures above 150 °C, thus enabling their use as waterborne wood adhesives with competitive dry/wet adhesive strength (5.4/3.5 MPa).^177^ Cationic lignin nanospheres function as activating anchors for hydrolases and enable aqueous ester synthesis by forming spatially confined biocatalysts upon self-assembly and drying-driven aggregation in calcium alginate hydrogel.^62^ Hao *et al*. developed a green pore-forming agent derived from lignin particles, which can be employed to prepare porous polysulfone (PSF) membranes *via* the phase inversion technique, thereby enhancing the performance of water treatment processes.^161^ Lignin particles with diameters ranging from 200 nm to 2.3 μm were converted into non-melting carbon nanospheres and microspheres, which serve as excellent CO_2_ capture agents with high mechanical strength and surface area. These materials are suitable for regeneration after multiple adsorption and desorption cycles.^178^ The formation of metal-phenolic network capsules was achieved by dissolving the central nucleus of lignin, which provides further evidence of the potential of lignin particles as templates for the modular insertion into the formation process of hollow superstructures by templated assembly. This study also demonstrated that the material has a good degradation rate.^4^ Overall, it is evident that lignin nanoparticles are evolving into versatile, green, advanced functional materials.

LNPs also face challenges that limit their potential end-users. A primary limitation stems from their compromised structural integrity in non-aqueous environments, attributed to their propensity for dissolution or colloidal aggregation when exposed to organic solvents or extreme pH conditions.^179^ This physicochemical instability significantly narrows the operational parameters for post-synthesis modifications and practical utilization. From a production standpoint, current synthesis protocols necessitate substantial volumes of aqueous and organic solvents, raising concerns regarding environmental sustainability and process scalability. While emerging research explores multifunctional applications, their integration into stimuli-responsive material systems remains in its nascent stages, requiring substantial innovation in surface engineering and hybrid material design to overcome current performance barriers.

## 4. Conclusions and future perspectives

A major challenge in lignin studies is the variability in lignin origins and extraction methods. This variability often leads to inconsistencies in research outcomes, making it difficult to compare results across different studies. The lignin polymerization strategy has the potential to address the issue of heterogeneity in industrial lignin feedstock. The current industrial lignin grades are mainly low molecular weight macromolecules that exhibit limited potential for high-performance materials, particularly those that require superior mechanical properties and chemical stability. Furthermore, the benzene rings impart high rigidity to lignin. It is a well-established phenomenon that polymers with higher molecular weights exhibit enhanced mechanical strength, thermal stability, flexibility, and other physicochemical properties. This indicates the broad potential for these polymers in a range of applications, including structural materials, biomedicine, electronic sensing, energy, and environmental applications. To expand the potential applications of lignin, it is essential to modify its molecular composition in addition to increasing molecular weight.

In general, the molecular weight, structure, and properties of copolymers can be precisely tuned by varying the ratios of monomers and the distributions of copolymer compositions. However, as a complex natural polymer, lignin's molecular weight, functional groups, and other structural properties are highly heterogeneous, which complicates the molecular modification process. This complexity manifests in two primary ways: (1) in terms of molecular structure, the abundance of functional groups provides numerous reactive sites for chemical modification, but also presents challenges in controlling lignin's macromolecular structure; (2) regarding the reaction system, free radical polymerization reactions are intimately tied to the solvent system. Due to the high degree of condensation of lignin, solubilization is often a limiting factor, which makes optimization of the reaction system both critical and challenging.

Although a multitude of avenues exists for the functionalization through chemical modification of lignin, avoiding chemical treatment should be preferred from a green chemistry point of view and as an effective strategy for reducing the overall costs associated with the process. Physical techniques for controlled supramolecular aggregation lignin permit the adaptable modulation of the material's micro- and nanoscale architecture, which can be employed independently or in conjunction with other modification methodologies to yield materials with specified morphologies and augmented value. In addition to physical and chemical modification approaches, enzyme-catalyzed modification and polymerization of lignin using initiation systems show promising prospects. Further work is also pivotal to shed light into the structure–function–performance triad of technical lignins with respect to their polymerization and application performance. With enhanced comprehension of the spatial and temporal dynamics of lignin polymerization, coupled with ongoing advancements in lignin chemistry, colloid chemistry, and polymer science, lignin is poised to become a sustainable, green bio-based raw material integral to human daily life and precision fields.

## Conflicts of interest

There are no conflicts to declare.

## Data Availability

No primary research results, software or code have been included and no new data were generated or analysed as part of this review.
